# Trace Oxygen Sensitive Material Based on Two Porphyrin Derivatives in a Heterodimeric Complex

**DOI:** 10.3390/molecules22101787

**Published:** 2017-10-21

**Authors:** Eugenia Fagadar-Cosma, Valentin Badea, Gheorghe Fagadar-Cosma, Anca Palade, Anca Lascu, Ionela Fringu, Mihaela Birdeanu

**Affiliations:** 1 Institute of Chemistry Timisoara of Romanian Academy, M. Viteazul Ave, No. 24, 300223 Timisoara, Romania; anca_palade@yahoo.com (A.P.); ancalascu@yahoo.com (A.L.); ionelacreanga@yahoo.com (I.F.); mihaione2002@yahoo.com (M.B.); 2 Faculty of Industrial Chemistry and Environmental Engineering, Politehnica University Timisoara, PtaVictoriei 2, 300006 Timisoara, Romania; valentin.badea@upt.ro; 3 National Institute for Research and Development in Electrochemistry and Condensed Matter, P. Andronescu Street, No. 1, 300224 Timisoara, Romania

**Keywords:** porphyrins, heterodimer complex, NMR spectroscopy, UV-vis spectroscopy, fluorescence, oxygen chemosensor

## Abstract

The successful preparation of a novel dimer complex formed between 5,10,15,20-tetrakis(3,4-dimethoxyphenyl)-porphyrin Fe(III) chloride and (5,10,15,20-tetraphenylporphinato) dichlorophosphorus(V) chloride using the well-known reactivity of the P–X bond is reported. The obtained complex was characterized by UV-vis, Fourier transform infrared spectroscopy (FT-IR), fluorescence, ^1^H-NMR, ^13^C-NMR, and ^31^P-NMR spectroscopic techniques and also by additional Heteronuclear Single Quantum Coherence (HSQC) and Heteronuclear Multiple Bond Correlation (HMBC) experiments in order to correctly assign the NMR signals. Scanning electron microscopy (SEM) and EDX quantifications completed the characterizations. This novel porphyrin dimer complex demonstrated fluorescence sensing of H_2_O_2_ in water for low oxygen concentrations in the range of 40–90 µM proving medical relevance for early diagnosis of diseases such as Alzheimer’s, Parkinson’s, Huntington’s, and even cancer because higher concentrations of H_2_O_2_ than 50 μM are consideredcytotoxic for life. Due to its optical properties, this novel metalloporphyrin–porphyrin based complex is expected to show PDT and bactericidal activity under visible-light irradiation.

## 1. Introduction

Sensing of oxygen has attracted particular interest because human beings can live maximally 10 min without supply of oxygen (the average need is 200 g/day) [1]. On the other hand, in order to realize personalized PDT treatment of cancer for each patient, in addition to the quality of the photosensitizer and of the light, molecular oxygen in irradiated tissues has to be severely limited and quantitatively monitored [2].

Besides biology, other fields requesting the monitoring of molecular oxygen are food packaging and environmental analysis due to the fact that aliments and many technical devices suffer from degradation in the presence of trace amounts of oxygen and water [3].

**Figure 1:**
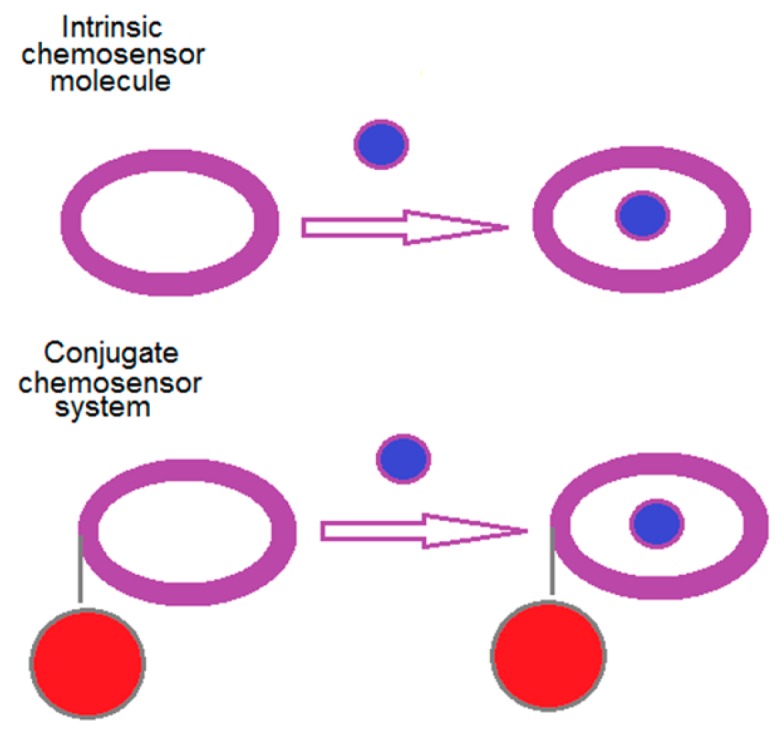
Fluorescence sensing system based on intrinsic ionophore with binding properties or conjugate with a fluorophore modifying the emission details.

A fluorescence sensing system comprises an ionophore, with function of binding, and a fluorophore that modifies the emission details [4]. Such a system can be composed by two distinct molecules or only one bearing two different functionalities, as represented in Figure 1. 

The main requirement for a chemosensing process is that the fluorophore has to display evident changes regarding optical recognition, either a significant change of color or regarding the intensity of light emission. The fluorophore might be bound to the receptor or simply located in the solution. 

Besides calixarenes and cyclodextrins, porphyrins [5] can be considered to be versatile synthetic receptors [6] having the capacity to coordinate or encapsulate guest molecules. Due to their ability to form amazing π-conjugated structures and to extend their aromaticity by tautomeric equilibria, porphyrins and metalloporphyrins deposited on various substrates are used for detection of either metal ions [7,8], anions [9,10,11], or gases [12,13] including molecular oxygen [14,15,16]. In the last decade, extensive ring fusion has become a novel target and Möbius aromatic molecules were reported [17] to be spontaneously generated.

Many new attempts to design novel axial bonding containing phosphorus and porphyrins have been performed in order to obtain advanced optoelectronic molecular assemblies with increased absorption on the whole visible light domain. In addition, due to their ability to generate singlet oxygen, various P(V) porphyrins were obtained [18,19] and investigated as photosensitizers [20,21].

Axial-bonding at phosphorus porphyrins generates two different types of complexes. If the pentavalent phosphorus atom is bonded to only two of the four nitrogen atoms of the porphyrin core, the sitting-atop (i-SAT) distorted complexes are generated. In this case the porphyrins preserve their two pyrrolic protons in the inner part of the macrocycle that can be evidenced by IR and H-NMR spectra [18].

The second case is the hexacoordinate cationic complex with two axial groups at the pentavalent phosphorus. The phosphorus atom is coordinated to all four nitrogen atoms in the porphyrin core, also involving the two pyrrolic protons in the complexation process [22]. It was demonstrated that the complex has a ruffed shape due to the fact that the phosphorus (V) ion is the smallest ion found to be inserted into a porphyrin core [23]. These phosphorus [P(POR)X_2_]^+^ cationic porphyrins that are not redox active at the central element can be linked to other compounds using the known substitution chemistry of the P–X bond.

Ferrocenyl functionalized porphyrin [24] complexes have been reported by axial bond formation involving phosphorus atoms [25] producing heterodimers and trimers [26] designed to mimic various natural processes. 

Extensive knowledge about the affinity of hemoglobin for oxygen, that is a function of pH and ionic strength, motivates us in our research, because signaling oxygen is required for monitoring of oxidative processes for medical purposes and for control of the environment in space habitable areas.

Derivatization of porphyrins containing phosphorus by novel axial bonding to peripheral groups of some metalloporphyrins represents an interesting approach to stereochemical tailoring of the structure and thus of chemical activity [27].

**Figure 2:**
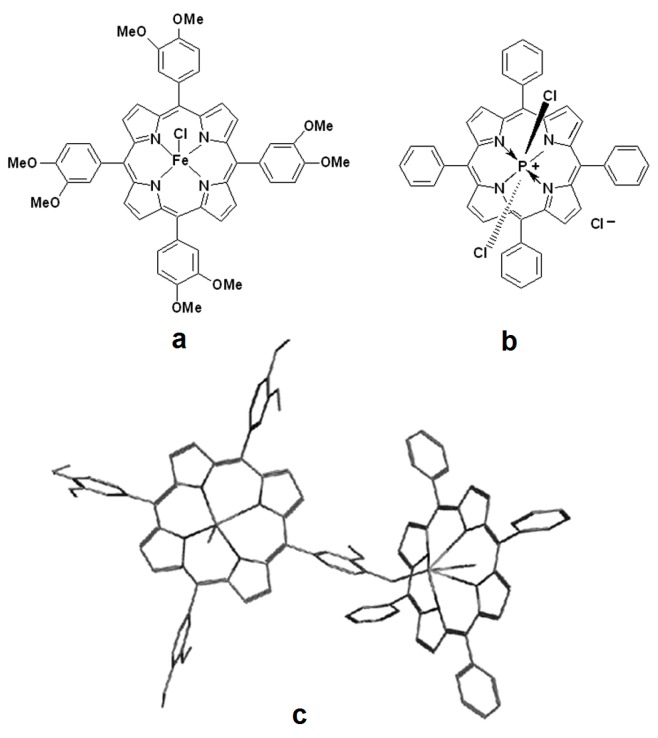
Fe(III) 5,10,15,20-tetrakis(3,4-dimethoxy-phenyl)-porphyrin chloride, porphyrin (compound **1**) (**a**), (5,10,15,20-Tetraphenylporphinato)dichlorophosphorus(V) chloride, porphyrin (compound **2**) (**b**) and the optimized structure of the porphyrin heterodimer complex (compound **3**) (**c**).

Based on our previous experience in synthesis, characterization, and applications of porphyrins [28,29,30] and also in dichlorophosphorus derivatives [31], in this paper we report successful preparation of a novel complex system formed between 5,10,15,20-tetrakis(3,4-dimethoxyphenyl)-porphyrin Fe(III) chloride (compound **1**) represented in Figure 2a and highly fluorescent (5,10,15,20-tetraphenylporphinato) dichlorophosphorus(V) chloride (compound **2**) shown in Figure 2b, using the well-known reactivity of the P–X bond. The initial porphyrin derivatives and the complex between Fe III metalloporphyrin and phosphorus (V) porphyrin were synthesized, characterized by UV-vis, fluorescence, FT-IR, ^1^H-NMR, ^13^C-NMR, and ^31^P-NMR spectroscopic techniques and also by additional Heteronuclear Single Quantum Coherence (HSQC) and Heteronuclear Multiple Bond Correlation (HMBC) experiments in order to assign correctly the NMR signals. The complex obtained from the FeIII-porphyrin and the phosphorus porphyrin was put into evidence by UV-vis monitoring. 

This novel complex demonstrated fluorescence sensing of H_2_O_2_ in water (the dependence between emission intensity and oxygen concentration is linear) for low oxygen concentrations in the range of 40–90 µM, thus proving medical relevance. This is the most important domain for medical detection, correlated with early diagnosis for diseases such as Alzheimer’s, Parkinson’s, Huntington’s, andcancer [32] because higher concentrations of H_2_O_2_ than 50 μM are considered cytotoxic for life [33].

This novel heterodimer complex (compound **3**), presented in Figure 2c, exhibiting improved fluorescence behavior, based on a non-fluorescent Fe-metalloporphyrin with already proven electrical O_2_ sensing capacities [34,35,36,37,38,39,40] and a highly fluorescent phosphor-porphyrin derivative is expected to act as conjugated chemosensor system for O_2_. These properties may also lead to potential future applications in biological fields, for example, as a bio-degradable ^1^O_2_-sensitizer [34] and to show photodynamic therapy of cancer (PDT) and bactericidal activity under visible-light irradiation [36,37,38,39].

## 2. Results and Discussions

### 2.1. NMR Characterization of 5,10,15,20-tetrakis(3,4-dimethoxy-phenyl)-porphyrin Fe(III) Chloride, Compound ***1***

#### 2.1.1. The ^1^H-NMR Spectrum of Compound **1**

The ^1^H-NMR spectrum of compound **1** presented in Figure S1 from Supplementary material is in accordance with the assigned structure including the shielding of *β* pyrrole protons, due to the diminution of the current of cycle and was already reported [40]. The presence of two methoxy groups asymmetrically disposed on each phenyl ring may distort the molecule and as a consequence the signals are more complicated (for example doublets instead of singlet). The protons from methoxy groups display a multiplet signal at 3.98–4.03 ppm; the four *meta* protons of phenyl presented a multiplet in the range 6.67–6.72 ppm; the eight *ortho* protons gave two singlet signals at 7.93 ppm and at 8.05 ppm, respectively and the *β*-pyrrolic protons display a singlet signal at 9.97 ppm. 

#### 2.1.2. The ^13^C-NMR Spectrum of Compound **1**

The ^13^C-NMR spectrum of compound **1**, shown in Figure S2 of the Supplementary material, evidenced the distinct chemical shift (δ) for the assignment of each type of carbon: The alkylic carbons from the eight CH_3_ groups display signals around 56–58 ppm, the *meso*carbons are resonating between 109.5 and 120.2 ppm, the *β*-pyrrolic carbons are resonating in the range of 127.4–134.8 ppm, the *α*-pyrrolic carbons give signals around 147 ppm and the phenylic carbons linked to OCH_3_ groups at about 150–153 ppm. The signal at 77 ppm is due to CDCl_3_ in which the sample was dissolved. Knowing that carbons without any attached hydrogen atoms give shorter signals, the signal height is also relevant, as illustrated in the case of substituted carbons in phenyl rings. On the other hand, symmetry duplication gave the same line but increases signal height. The ^13^C DEPT135 spectra provided data for identifying the types of carbons in a molecule by differentiating them on the basis of number of attached protons. Carbons without attached protons are not observed. The carbons of CH_3_ and CH groups are pointing upwards, instead, the methylene carbons (CH_2_) are pointing downwards. 

### 2.2. NMR Characterization of (5,10,15,20-tetraphenylporphinato)dichlorophosphorus (V) Chloride, Compound ***2***

#### 2.2.1. The ^1^H-NMR Spectrum of Compound **2** (Figure S3 from Supplementary Material)

It is already known that the porphyrin ligand might adopt nonplanar conformations in response to steric or electronic effects induced by the nature of the central coordinated atom and the axial ligands. Many previous reports are dealing with distortion of structure for the complexes between free base meso-tetraarylporphyrins and various σ-acceptors, such as: Tetracyanoethylene [41], trialkylsilyl chlorides [42], uranyl salts [43], and zirconium (IV) chloride [44,45]. According to [46], the generation of the phosphorus complex determines the four pyrroles to corrugate and locate alternatively up and down relative to the metalloporphyrin plane, thus the two nitrogen atoms realizing a better position for coordination of to the phosphorus acceptor [47]. In a previously reported paper [48], the authors were discontent by the fact that the presence of a singlet signal for the *β*-protons in the ^1^H-NMR spectra of the complexes does not correspond to the asymmetrical pyrrole rings in the proposed structure. As it can be seen in our ^1^H-NMR spectrum, even if the ring inversion of the tilted core conformation of the porphyrin is fast, the β-protons appear as two distinct doublet signals both upfield shifted to 8.88 and 9.14 ppm, completely illustrating the lack of symmetry. Comparing the ^1^H-NMR spectra of the tetraphenylporphyrin (TPP) with that of compound **1**,the *ortho*-phenyl protons of the second are downfield shifted.

The absence of the absorption band at 3320 cm^−1^ assigned to the N–H stretching vibration in the FT-IR spectrum of compound **2** (Figure S4 from Supplementary Material) corroborated with the missing resonance for the N–H protons around (−2.70 ppm), in the ^1^H-NMR spectrum (Figure S3b from Supplementary material) confirmed the proposed structure for the hexacoordinated phosphorus (V) porphyrin, compound **2**.

#### 2.2.2. The ^13^C NMR Spectrum of Compound **2**

In the ^13^C NMR spectrum of compound **2** (Figure S5 from supplementary material) more sharp signals were displayed at 117 ppm assigned to *meso* C, 127 ppm, assigned to meta carbons, 128 ppm assigned to para carbons, 132 ppm corresponding to *β* carbons, 134 ppm assigned to *ortho* carbons, and 139 ppm assigned to phenyl C-linked in *meso* position.

#### 2.2.3. The ^31^P-NMR Spectrum of Compound **2**

The ^31^P-NMR spectrum of compound **2** confirms the coordination of phosphorus(III) chloride to the porphyrins (Figure S6 from Supplementary Material). The proton-decoupled ^31^P-NMR (202.4 MHz, CDCl_3_) spectra (85% H_3_PO_4_ external reference) gave signals at −228 ppm (s, outer P(V)porphyrin) and −193.22 (s, inner P(V) ppm for the PCl_3_–porphyrin complex.

#### 2.2.4. 2D Spectra ^1^H-^13^C, HSQC, and HMBC Experiments for Compound **2**

For the complete assignation of NMR signals, 2D spectra ^1^H-^13^C, HSQC, and HMBC experiments for compound **2** (Figure S7 from Supplementary material) were performed and confirmed the distorted structure.The HSQC ^1^H-^13^C (Heteronuclear Single-Quantum Correlation) spectra gives direct one-bond correlations between carbons and protons.

An interaction between the *β*-protons and the group of C signals in the range 132.5–133.5 ppm determined the correct assignment of the *β* carbons. *Ortho* protons are also in interaction with *ortho* carbons that are correctly assigned at 134 ppm.

After identifying single bond correlations, we pass to assign HMBC which offer tremendous information about 2 and 3 bond CH coupling. The HMBC ^1^H-^13^C (Heteronuclear Multiple Bond Correlation) spectra gives correlations between carbons and protons that are separated by two, three, and, sometimes in conjugated systems, four bonds. Direct one-bond correlations are suppressed. The intensity of cross peaks depends on the coupling constant, which for three-bond couplings follows the Karplus relationship. For dihedral angles near 90 degrees, the coupling is near zero. Thus, the absence of a cross peak does not confirm that carbon–proton pairs are many bonds apart. The most evident are the 2–3 bond interactions between *β*-protons and *meso* carbons and the four bond interaction between *ortho* protons and *β*-carbons. *Para* carbons are also interacting with *ortho* protons.

### 2.3. Physical Characterization of the Dimer Complex, Compound ***3***

#### 2.3.1. The ^1^H-NMR Spectrum of the Dimer Complex Compound **3**

**Figure 3:**
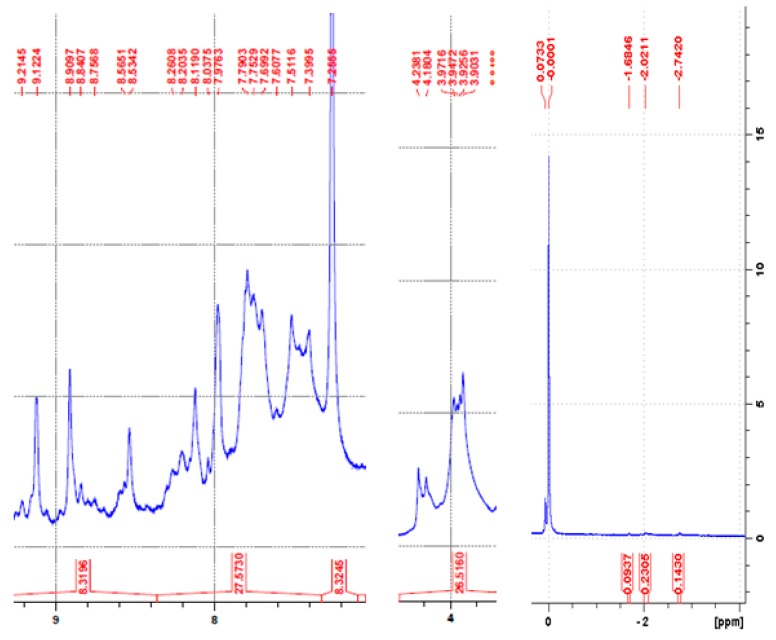
^1^H-NMR spectrum of the complex compound **3**.

^1^H-NMR spectrum of the dimer complex compound **3** does not show any signal of internal NH, as expected (Figure 3). The differences in the number of protons resulting from integrating ^1^H-NMR *meso*- situated methoxy signals in comparison with *para*-positioned methoxy signals can be correlated with the^31^P-NMR spectrum in which the signal from −99.3 ppm can be attributed to P–O–Aryl bonding [46] and the conclusion is that the product is formed by linking one molecule of P(V) porphyrin (compound **2**) with one molecule of Fe(III)-porphyrin compound **1** by P–O–Aryl bonding due to favorable presence of AgNO_3_ and well established great affinity of phosphorus to oxygen [47].

The interaction of *meso*-tetra-(3,4-dimethoxy-phenyl) porphyrinato Fe(III)-(compound **1**) with P(V)-porphyrin-(compound **2**) produced P(V)-porphyrin-Fe(III)-porphyrin complex-(compound **3)** with the molar ratio of 1:1 (donor:acceptor). ^1^H-NMR spectrum of the complex compound **3** indicated that in the prepared molecular complex, the central core of the Fe(III) porphyrin (compound **1**) was corrugated and further linked to functional *para*-methoxy group in the metalloporphyrin, acting as σ-electron donor to phosphorous atom from compound **2**. The integrated number of protons shows that the number of *meta*-OCH_3_ protons is higher than the number of *para*-OCH_3_ protons, proving the involvement of only *para*-OCH_3_ group in the new bond formation.

#### 2.3.2. ^31^P-NMR Spectrum of the Heterodimer Compound **3**

^31^P-NMR spectrum of the heterodimer complex compound **3** makes it possible to distinguish between signals coming from the inner and outer surfaces (Figure S10 from Supplementary data). The signals are located at −227.8 ppm, characteristic to outerP(V) atoms, at −190.3 and −192.8 ppm both assigned to inner P(V) porphyrinand a signal at −99.3 ppm, characteristic to phosphorus in P–O–aryl groups.

**Figure 4:**
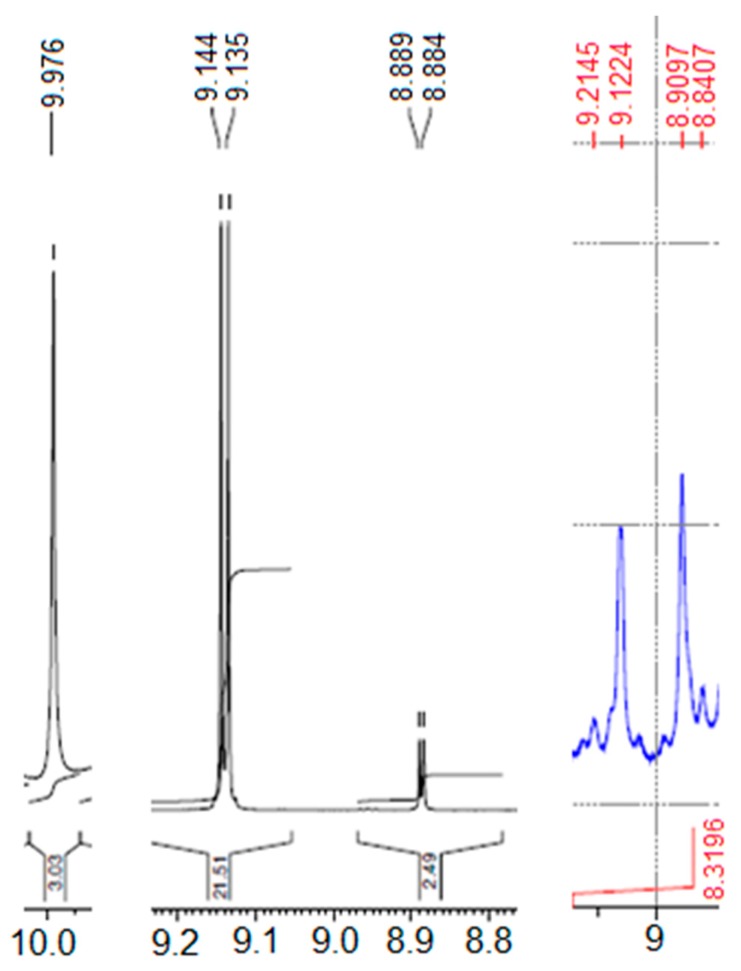
Differences between the *β* signals in ^1^H-NMR spectroscopy between Fe (III) porphyrin (compound **1**), P(V) porphyrin (compound **2**) and the heterodimer complex compound **3** (presented in this order).

The main differences between the *β* signals in NMR spectroscopy between Fe (III) porphyrin, P(V) porphyrin and the complex system of two porphyrins are presented in Figure 4. As can be seen, the Fe-metalloporphyrin (compound **1**) with increased symmetry has all *β* protons equivalents, in distorted P(V) porphyrin (compound **2**) there are two distinct doublet like signals and in the dimer complex compound **3** two distinct multiplet signals are displayed.

**Figure 5:**
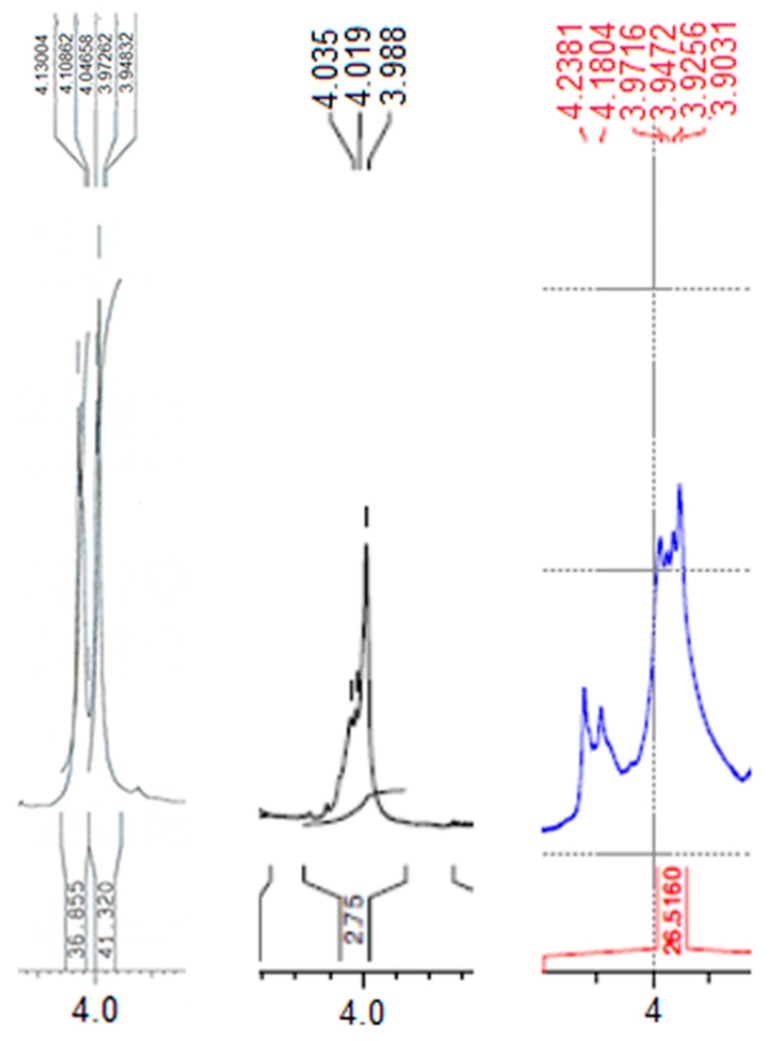
Differences between the protons from methoxy signals in ^1^H-NMR spectroscopy between 5,10,15,20-tetra(3,4-dimethoxy-phenyl)-porphyrin base, Fe (III) porphyrin (compound **1**) and the dimer complex compound **3**.

Figure 5 presents the important differences between the protons from methoxy signals in NMR spectroscopy between 5,10,15,20-tetra(3,4-dimethoxy-phenyl)-porphyrin, that is the porphyrin base of Fe (III) porphyrin, Fe (III) porphyrin (compound **1**), and the dimer complex compound **3**. Regarding the ^1^H-NMR of the porphyrin base, all four methoxy groups in the 4 position have all 12 protons equivalents and also all four methoxy groups in the 3 position display an equal number of 12 equivalent protons. In the case of the more symmetrical Fe(III) porphyrin (compound **1**), all the 24 protons are equivalent, but in the case of the dimer compound **3**, the number of protons in the methoxy groups from *para*-position displayed as doublet signal at 4.18–4.23ppm has a reduced number of protons in comparison with the number of protons from *meso*-position giving signal as multiplet in the range of 3.90–3.97ppm, meaning that P(V) porphyrin is totally linked to the oxygen atom from *para-*OCH_3_ position of Fe-metalloporphyrin (compound **1**) (as presented in Figure 2c).

EDAX Quantification (Figure S11 from Supplementary material) reveals, in strong agreement with NMR data, that in the heterodimer complex compound **3** to a Fe atom is corresponding one P atom. 

#### 2.3.3. The FT-IR spectrum of compound **3**

**Figure 6:**
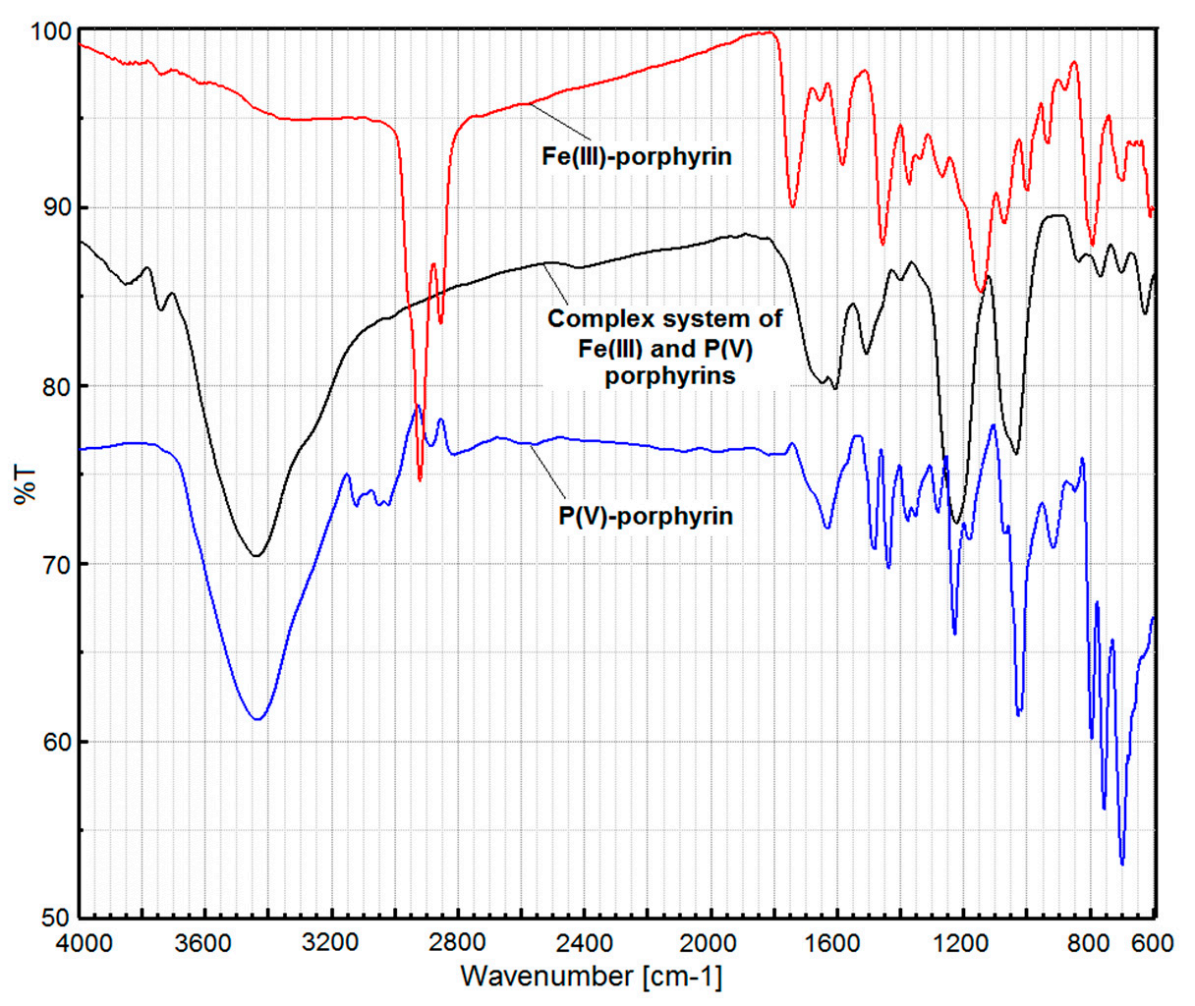
The overlapped FT-IR spectra of Fe(III)porphyrin (compound **1**), P(V) porphyrin (compound **2**) and their heterodimer complex (compound **3**).

The FT-IR spectrum of complex formed between compound **1** and compound **2** (Figure 6, see also detailed Figure S8 in Supplementary data) showed the very sharp band assigned to POC aromatic at 1222 cm^−1^ and its additional intense band located at 1033 cm^−1^ accompanied by weak band at 836 cm^−1^ undoubtly assigned to P–O single streching vibration [48].

### 2.4. UV-Vis Analysis

#### 2.4.1. The UV-Vis Study Regarding the Behavior of the Compound **2** in Solvents of Different Polarity

**Figure 7:**
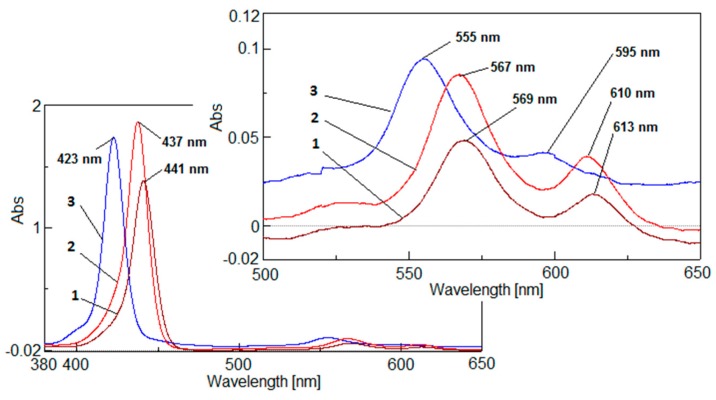
UV-vis spectra of compound **2** in H_2_O (line 1), DMF (line 2), and acetone (line 3).

The UV-vis study regarding the behavior of the compound **2** in solvents of different polarity (Figure 7) showed that the higher is the solvent polarity, the stronger the bathochromic effect of the Soret and Q bands is. This red shift can be explained by the enhanced effect on the charge separation state in the cationic P(V) porphyrin.

#### 2.4.2. UV-Vis Monitoring of the Compound **3** Generation

**Figure 8:**
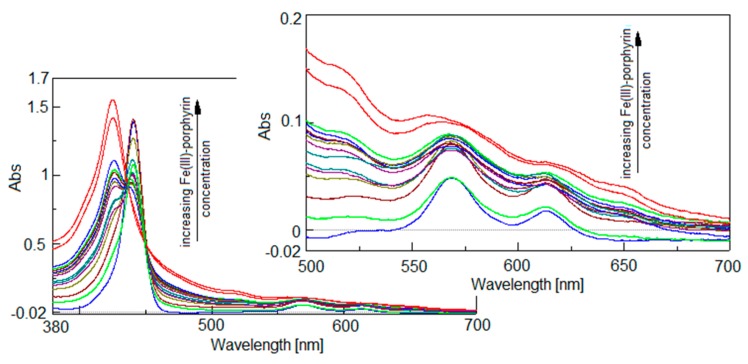
Monitoring of the complex compound **3** formed between compound **1** and compound **2**.

The heterodimer complex compound **3** formed between compound **1** and compound **2** was monitored by registering the UV-spectra (Figure 8), which display two clearly defined isosbestic points located at 437 and 450 nm that certify the reactions equilibria for intermediate products formed during P-*O*-alkyl linking. A double interaction can take place as follows: The central core of the Fe(III) porphyrin, compound **1**, was corrugated and one of the functional *p*-methoxy groups of the metalloporphyrin acted as σ-electron donor to phosphorous atom from P(V)porphyrin, compound **2**. Formation of the dimer complex compound **3** causes the pyrroles to corrugate and locate alternatively up and down the metalloporphyrin plane, generating tautomeric species put into evidence by the two clearly defined isosbestic points in UV-vis spectra.

**Figure 9:**
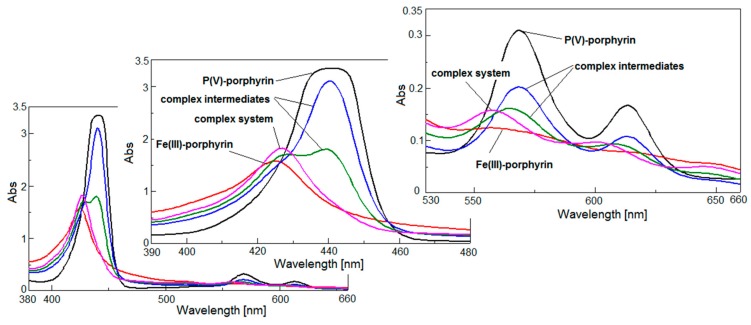
Overlapped UV-vis spectra of the initial Fe(III) porphyrin (compound **1**) and P(V) porphyrin (compound **2**) the intermediate reaction species and the dimer complex compound **3**. Details of Soret and Q bands.

Figure 9 presents the evolution of the main species in UV-vis during the synthesis of the porphyrin dimer complex. As can be seen, the final heterodimer complex compound **3** has the Soret band strongly hypsochromically shifted in comparison with compound **2**, but slightly bathochromically shifted in comparison with compound **1**. The most important aspect of the dimer complex compound **3** UV-vis spectrum is related with the location of the Q bands that are both hypsochromically shifted in comparison with the Q bands of the two initial porphyrin precursors, but hyperchromic and better shaped in comparison with the Fe-metalloporphyrin.

### 2.5. Fluorescence Properties

**Figure 10:**
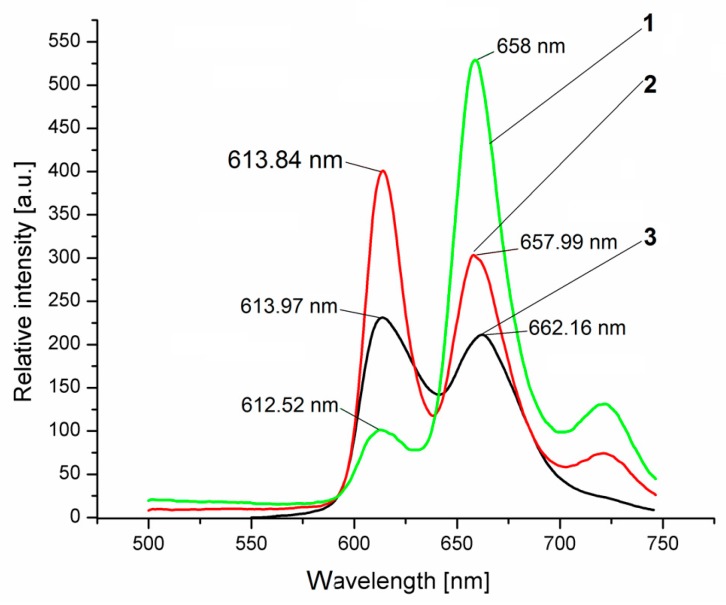
Emission spectra of dimer complex compound **3** treated with H_2_O_2_ (line 1), of bare complex compound **3** (line 2), and of P(V) porphyrin (compound **2**) (line 3).

Because we believed that we obtained a new conjugate chemosensor system that left the iron core of the dimer complex free to interact with oxygen molecules, fluorescence properties of these porphyrin derivatives have been studied in comparison and also after treatment with H_2_O_2_. The emission spectra of P(V) porphyrin (compound **2**) in DMF (Figure 10) exhibited only two unresolved maxima similar in intensity at 614 and 662 nm, probably belonging both to theQ_x_ (0,0) fluorescence band. In the emission spectra, the dimer complex compound **3** displays three bands, with higher intensity in the same region with compound **2**, accompanied by a weaker emission band located at 721 nm, assigned to Q_x_(0,1) transition both before and after treatment with H_2_O_2_.

Exposure to H_2_O_2_ produces significant changes regarding the shape of the emission spectrum. A double increase of the intensity of the band located at 658 nm is taking place and it is accompanied by a significant decrease of the band located at 612 nm. All these porphyrin derivatives can be considered in the class of second generation photosensitizers, exhibiting emmision at λ > 630 nm.

**Figure 11:**
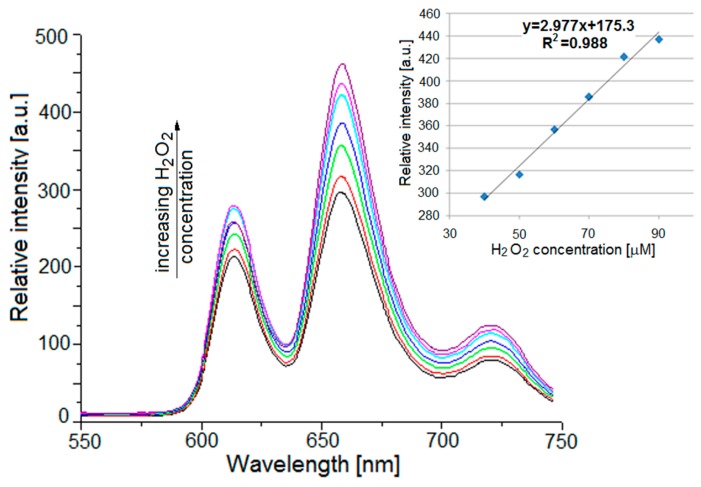
Emission spectra of dimer complex compound **3** aftertreatment with H_2_O_2_ and the linear dependence of the intensity of the band located at 658 nm with increasing of H_2_O_2_ concentration.

This situation and the fact that the Fe atom from the dimer structure remains free to interact with oxygen, encouraged us to study the fluorescence behavior of compound **3** when exposed to increased concentrations of H_2_O_2_ in a DMF-water system. By continuously monitoring the changes in intensity of the band from 658 nm, produced by the exposure to increased concentrations of H_2_O_2_, we noticed that the intensity of the band increased as the H_2_O_2_ concentration increased, the linear trend of the response and the very good correlation coefficient are shown in Figure 11.

The detection of H_2_O_2_ is not based on fluorescence quenching of the sensing assembly, but on increasing of the emission intensity.

**Scheme 1:**
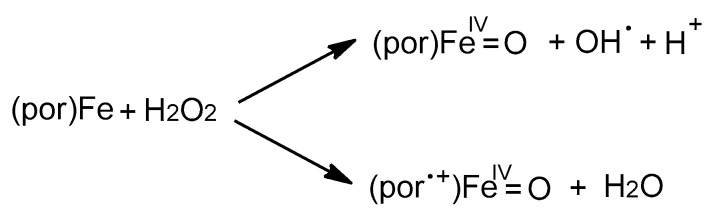
The mechanism of hydrogen peroxide decomposition by a Fe-porphyrin.

The mechanism of reaction was already established and is based on formation during the reaction with hydrogen peroxide of higher oxidation states of intermediate iron porphyrins. The structure of these intermediates, partially confirmed by our UV-vis experiments (as seen in Figure S12 from supplementary file), are according to Scheme 1: Oxoferryl species (por)-Fe**^IV^** = O [or its protonated form (por)Fe**^IV^**–OH] formed by homolysis of the peroxide and oxoperferryl species (por^•+^)Fe**^IV^** = O formed by heterolysis [49,50,51,52,53]. The UV-vis study (Figure S12) demonstrates the existence of both oxo-iron (IV) porphyrin species (the shifted band at 520 nm) and of the oxo-iron-porphyrin radical cation species (the isosbestic points at 450 nm and for a few number of curves at 505 nm).

### 2.6. Microscopic Analysis

**Figure 12:**
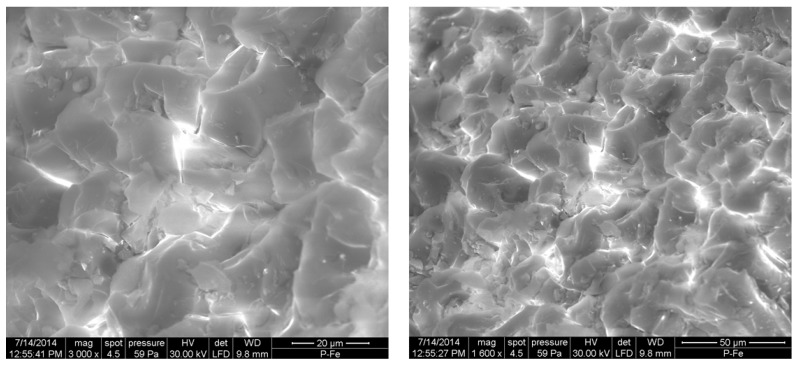
Scanning electron microscopy (SEM) images of the platelet like crystals of Fe-porphyrin (compound **1**).

**Figure 13:**
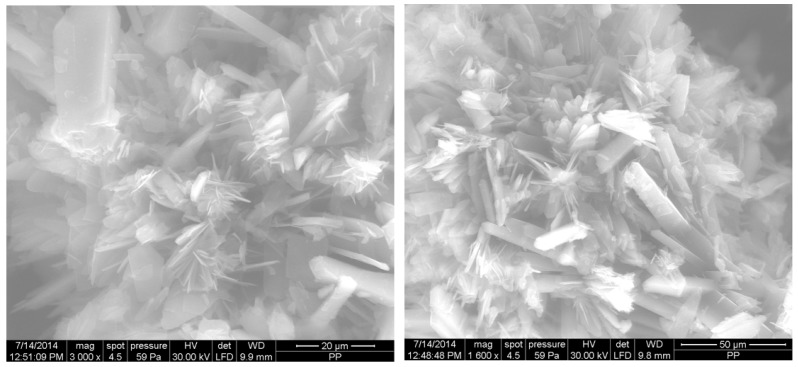
SEM images of the needle like crystals in case of water-soluble P(V)porphyrin (compound **2**).

**Figure 14:**
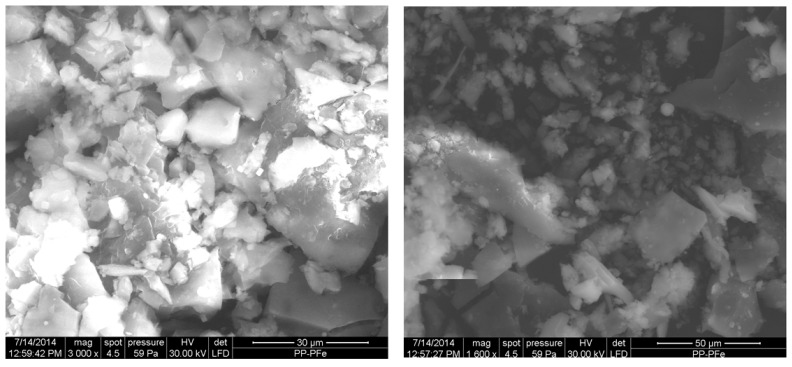
SEM images of the inhomogeneous grains of the new heterodimer complex compound **3**.

SEM images of the surfaces are shown in Figure 12, Figure 13 and Figure 14 and reveal multilayer platelet-like crystals in case of compound **1**, needle-like crystals of various sizes in case of water-soluble porphyrin compound **2,** and inhomogeneous grains from several micrometers up to 25 micrometers (several of the larger presenting cubic shape) in case of the new dimer complex compound **3**. The electron micrographs do not put into evidence an easily recognizable crystalline structure for the complex.

## 3. Materials and Methods

### 3.1. Materials

All reagents were p.a. grade and provided by Sigma Aldrich (Darmstadt, Germany), Alpha-AESAR (Karlsruhe, Germany), and Merck (Darmstadt, Germany), and were used as received. Chloroform was previously stored over 4Å molecular sieves and CH_2_Cl_2_ was distilled from CaH_2_ (nitrogen atmosphere).

### 3.2. Syntheses

#### 3.2.1. Method for Synthesis of Compound **1**

The metalloporphyrin, 5,10,15,20-tetrakis(3,4-dimethoxy-phenyl)-porphyrin Fe(III) chloride, compound **1**, represented in Figure 2a, was obtained by adapting the procedure of metallation of the porphyrin base described in literature [54,55] using large excess of iron (III) chloride [40].

#### 3.2.2. Method for Synthesis of Compound **2**

(5,10,15,20-Tetraphenylporphinato)dichlorophosphorus(V)chloride, compound **2**, was prepared modifying the method reported by Carrano [56,57] by reaction of tetraphenylporphyrin (123 mg, 0.2 mmol) with large excess of POCl_3_ (2 mL, 21.85 mmol) in 20 mL dry pyridine, under reflux in N_2_ atmosphere (until UV-vis monitoring showed that the Soret and Q bands were changed) followed by pyridine evaporation under high vacuum. The residue was dissolved in CH_2_Cl_2_, filtered, and the filtrate was washed with diluted HCl, dried, and evaporated. Column chromatography on neutral alumina with chloroform containing 1% formic acid, followed by recrystallization from CH_2_Cl_2_/hexane*v*/*v* = 1/1 gave water soluble dark violet needles. Corroborating the fact that the color of the mixture changed from dark violet to dark green with ^1^H-NMR and UV-vis spectral data provided proof that the porphyrin core suffers a deformation process.

For comparison, we have also synthesized a small amount of compound **2** by the method recently published by M Senge [21]. According to this method, pentavalent phosphorus was inserted into porphyrin by reaction of porphyrin base with large excess of PCl_3_ and of 2,6-lutidine (molar ratio 1/15/115) in dry dichloromethane under argon atmosphere. The main product, compound **2**, was formed due to the air oxidation during the reaction.

The third attempt to obtain compound **2** was by using TPP and PCl_3_ in molar ratio of 1/1, with grinding at ambient temperature for 30 min, in solvent free conditions [53,58]. 

#### 3.2.3. Method for Synthesis of Compound **3**

The porphyrin dimer complex compound **3** (Figure 2c) was obtained by refluxing together, for two hours a solution comprised of 1.45 mg (1.93 × 10^−3^ mole) compound **2** in 10 mL DMF and 1.86 mg (1.97 × 10^−3^ mole) compound **1** dissolved in 10 mL DMF in the presence of AgNO_3_ to favor P–O bond formation. The pure alkoxy heterodimer derivative was easily separated by column chromatography on alumina with chloroform as an eluent.

Taking into account the analysis results and the ability of substituted porphyrins to form different chair- and table-like conformers [59], the following structure, as presented in Figure 2c, was proposed for the complex. The access to Fe to be further engaged in bonding of oxygen is allowed by this synthetic modification, making it also an attractive structure for gas storage.

#### 3.2.4. The Main Characteristics of the Heterodimer Complex Compound **3**

The main characteristics of the heterodimer complex compound **3** (Figure 2c) are: dark red crystals; yield 89%; mp over 320 °C ^1^H-NMR (CDCl_3_, 500MHz), d, ppm: 3.90–3.97 (m, 12H, 3-OCH_3_), 4.18–4.23 (m, 9H, 4-OCH_3_), 7.39–7.51 (m, 4H, m-Ph), 7.60–7.79 (m, 8H, m-Ph), 7.97–8.03 (m, 8H, o-Ph), 8.11–8.26 (m, 4H, p-Ph), 8.53–8.56 (m, 8H, o-Ph), 8.90(m, 8H, β-Pyr), 9.12(m, 8H, β-Pyr). ^31^P-NMR (CDCl_3_, 202 MHz) δ(ppm): −227.8 (outer P(V)); −190.3, 192.8 (inner P(V)); −99.3 (P-O-Ar), FT-IR complex (ATR) cm-1: 767 (w, δC-H p-substituted Ph); 837 (w, νP-O); 1034 (i, νP-O, νC-N); 1223 (i, νP-O-Car); 1606–1648 (m, νC=C); UV-vis, DMF (λ_max_ (log ε)): 427 (5.58); 517 (4.50); 557 (4.51); 601 (4.27).

### 3.3. Apparatus

UV-vis spectra were recorded in the 380–700 nm range on a UV-vis JASCO V-650 apparatus (Hachioji, Tokyo, Japan). FTIR spectra were recorded on a JASCO 430 FT-IR spectrophotometer (JASCO Labor-und Datentechnik GmbH, Gross-Umstadt, Germany), by using KBr pellets, in the 400–4000 cm^−1^ range or using ATR module. NMR spectra were recorded on a BrukerAvance III 500 (500.0 MHz for ^1^H, 125.0 MHz for ^13^C) and 202.4 MHz for ^31^P) spectrometer (Bruker, Karlsruhe, Germany), using TMS as internal reference and CDCl_3_ as solvent. Phosphorus chemical shift values are given in ppm with 85% H_3_PO_4_ as external standard, with positive numbers indicating a downfield shift. The morphology of the sample particles wasinvestigated by field emission-scanning electron microscopy (SEM)/energy dispersive X-ray spectroscopy EDX, Model INSPECT S, (Electronics Yorkshire, Airedale House, Leeds, UK). MS investigation was performed on a Bruker esquire HCT series mass spectrometer (Bruker Daltonics, Bremen, Germany), with Atmospheric Pressure Interface-ElectroSpray Ionization. Fluorescence spectra were recorded in DMF on a Perkin-Elmer Model LS 55 apparatus (PerkinElmer, Inc. /UK Model LS 55, Waltham, MA, USA). The emission spectraof all the compounds were registered at room temperature, in dilute solutions (10^−7^ mol/L) with the aim to minimize the self-quenching effect [60] in a 1cm × 1cm optical quartz cell exciting at λ_ex_ = 430 nm (the Soret band, to have better signal to noise ratio) using slit widths, 10 for excitation and 5 for emission. 

The porphyrin’s starting structure was drawn with ChemDraw 10.0 tool (PerkinElmer Informatics-Cambridge Soft Corporation, Cambridge, MA, USA), and was converted to a 3D structure using Discovery Studio from Accelerys (San Diego, CA, USA). The image was capture using the Pymol module from Schrodinger. (Accelrys Software, Inc., Discovery Studio Modeling Environment, Release 4.0, San Diego, CA, USA, 2013).

The PyMOL Molecular Graphics System, Version 1.7.4 Schrödinger, (LLC New York, NY, USA, 2015).

## 4. Conclusions

In this paper, we report successful preparation of a novel conjugate chemosensing system for O_2_ detection formed between non-fluorescent 5,10,15,20-tetrakis(3,4-dimethoxyphenyl)-porphyrin Fe(III) chloride (compound **1**) and highly fluorescent (5,10,15,20-tetraphenylporphinato) dichlorophosphorus(V) chloride (compound **2**), by using the well-known reactivity of the P–X bond. The initial porphyrin derivatives and the complex **3** formed between Fe III metalloporphyrin (compound **1**) and Phosphorus (V) porphyrin (compound **2**) were synthesized, characterized by UV-vis, fluorescence, FT-IR, ^1^H-NMR, ^13^C-NMR, and ^31^P-NMR spectroscopic techniques, and also by additional 2D spectra ^1^H-^13^C, HSQC, and HMBC in order to correctly assign the NMR signals. The formation of the dimer complex, compound **3,** was put into evidence by UV-vis monitoring. A double interaction can take place as follows: the central core of the Fe(III) porphyrin (compound **1**) was corrugated and one functional *p*-methoxy group in the metalloporphyrin acted as σ-electron donor to phosphorous atom from P(V)porphyrin (compound **2**). Formation of the complex compound **3** causes the pyrroles to corrugate and locate alternatively up and down the metalloporphyrin plane, generating tautomeric species put into evidence by the two clearly defined isosbestic points in UV-vis spectra.

SEM images of the surfaces reveal multilayer platelet-like crystals in case of compound **1**, needle-like crystals of various size in case of water-soluble phosphorus porphyrin compound **2**, and inhomogeneous grains from few micrometers up to 25 micrometers (several of the larger presenting cubic shape) in case of the new dimer complex compound **3**.

This novel heterodimer complex compound **3**, that preserves the iron core unchanged properties, demonstrated fluorescence sensing of H_2_O_2_ in DMF–water environment for low concentrations in the range of 40–90 µM, proving medical relevance for early diagnosis of diseases such as Alzheimer’s, Parkinson’s, Huntington’s, and even cancer because concentrations of H_2_O_2_higher than 50 μM are considered cytotoxic for life. Due to its fluorescence properties extended to 750 nm, this novel metalloporphyrin-porphyrin based complex compound **3** will also find future application in PDT asasecond-generation photosensitizer and in bactericidal trials under visible-light irradiation.

## Supplementary Materials

The supplementray materials Figures S1–S12 are available online.
