# Supplementary material for: Trace Oxygen Sensitive Material Based on Two Porphyrin Derivatives in a Heterodimeric Complex
Source: Molecules. 2017 Oct 21;22(10):1787. doi: 10.3390/molecules22101787 (PMC6151409; doi:10.3390/molecules22101787)
Supplement: Supplementary file 1 [file molecules-22-01787-s001.pdf]

Trace oxygen sensitive material based on two porphyrin derivatives heterodimer complex

Eugenia Fagadar-Cosma*[,a], Valentin Badea [b], Gheorghe Fagadar-Cosma*[,b], Anca Palade [a], Anca Lascu [a], Ionela Fringu [a], Mihaela Birdeanu[a,c]

[a] *Institute of Chemistry Timisoara of Romanian Academy, M. Viteazul Ave, No. 24, 300223-Timisoara, Romania, e-mail: efagadar@yahoo.com, fax: +40-256-491824, tel: +40-256-491818*
[b] *Politehnica University Timisoara, Faculty of Industrial Chemistry and Environmental Engineering, Pta Victoriei 2, 300006-Timisoara, Romania, e-mail: gfagadar@yahoo.com, fax: +40-256-403021,*
tel: +40-256-403000
[c] *National Institute for Research and Development in Electrochemistry and Condensed Matter, P. Andronescu Street, No. 1, 300224- Timisoara, Romania.*

1. The ^{1}H-NMR spectrum of 5,10,15,20-tetrakis(3,4-dimethoxy-phenyl)-porphyrin Fe(III) chloride, compound **1**

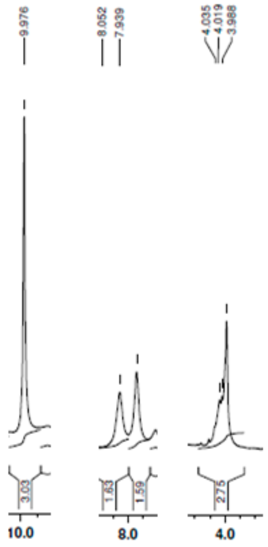

S1 The ^{1}H-NMR spectrum of 5,10,15,20-tetrakis(3,4-dimethoxy-phenyl)-porphyrin Fe(III) chloride, compound **1**

2. The ^{13}C NMR spectrum of 5,10,15,20-tetrakis(3,4-dimethoxy-phenyl)-porphyrin Fe(III) chloride, compound **1**

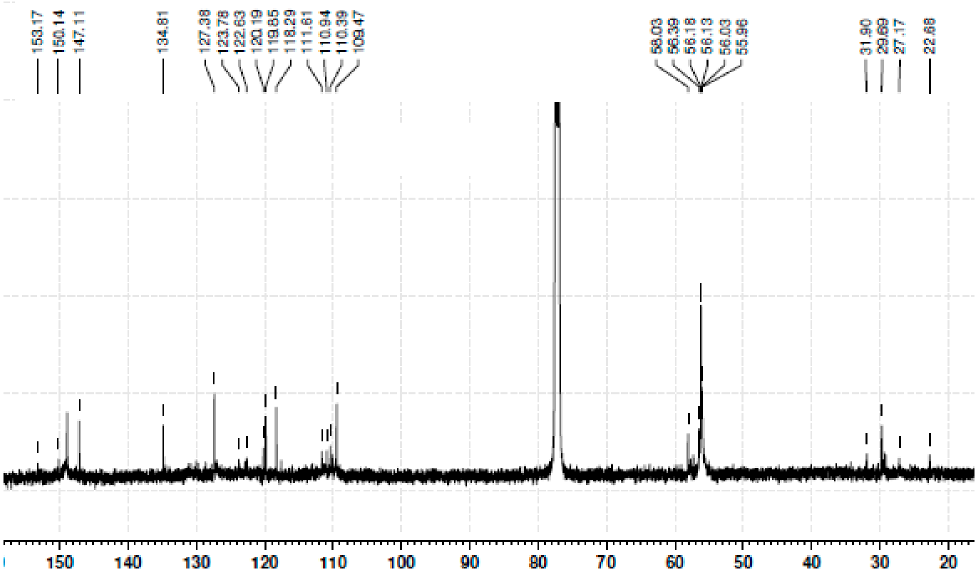

S2 The ^{13}C NMR spectrum of 5,10,15,20-tetrakis(3,4-dimethoxy-phenyl)-porphyrin Fe(III) chloride, compound **1**

3. The ^{1}H-NMR spectrum of (5,10,15,20-Tetraphenylporphinato)dichlorophosphorus (V) chloride, compound **2**

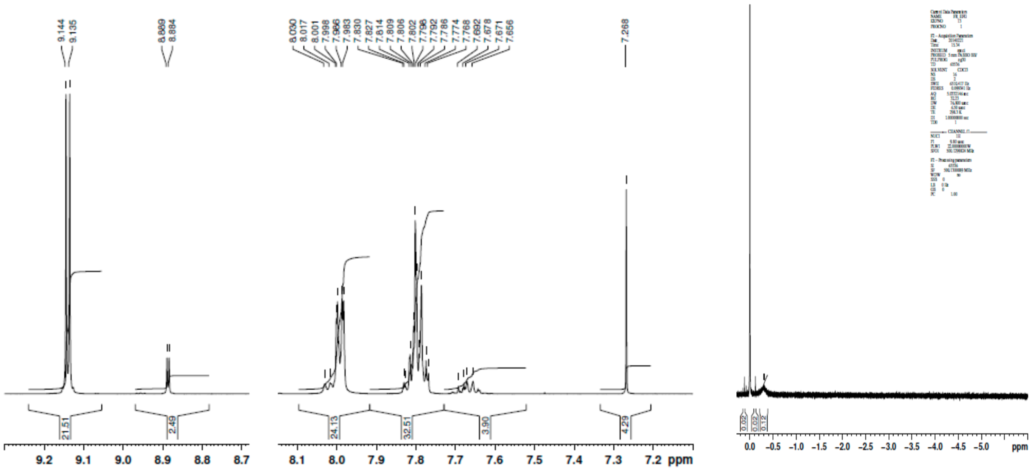

a) b)

S3 The ^{1}H-NMR spectrum of (5,10,15,20-Tetraphenylporphinato)dichlorophosphorus (V) chloride, compound **2**. (a) The ^{1}H-NMR (detail of range 0 ÷ -5ppm) spectrum of (5,10,15,20-Tetraphenylporphinato)dichlorophosphorus (V) chloride, compound **2** proving the fact that there is no internal NH proton (b)

4. FT-IR spectrum of compound **2**

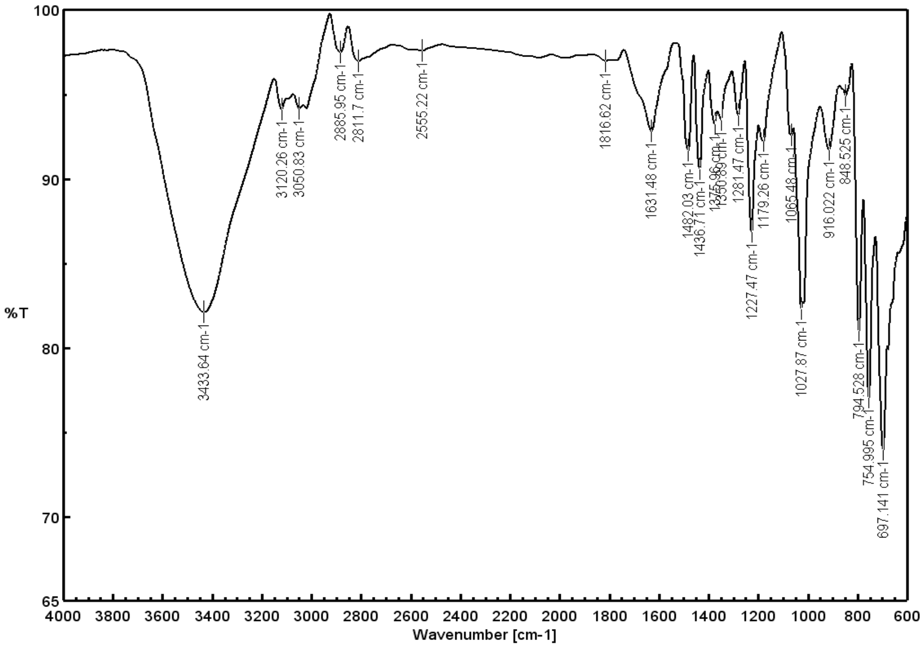

S4 The FT-IR spectrum of (5,10,15,20-Tetraphenylporphinato)dichlorophosphorus (V) chloride, compound **2**

5. ^{13}C-NMR spectrum of (5,10,15,20-Tetraphenylporphinato)dichlorophosphorus (V) chloride, compound 2

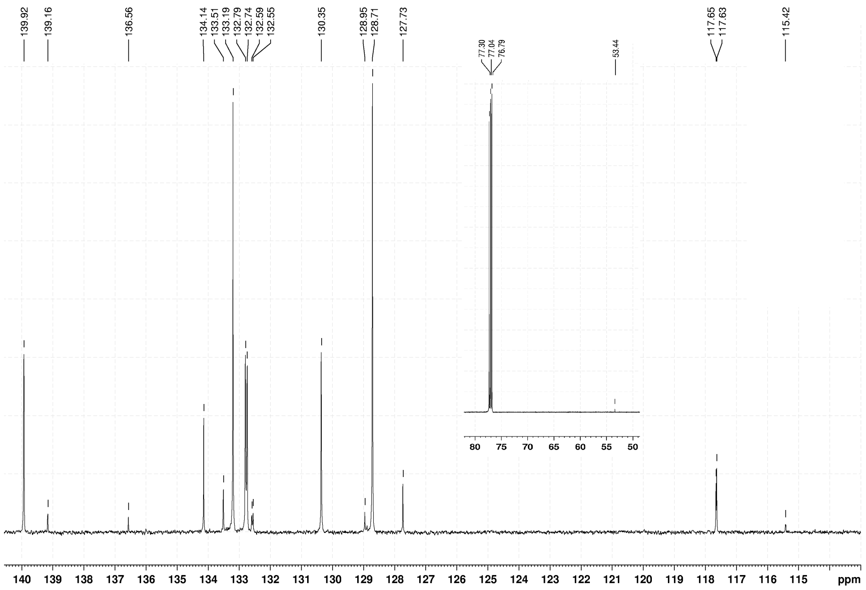

S5 The ^{13}C-NMR spectrum of (5,10,15,20-Tetraphenylporphinato)dichlorophosphorus (V) chloride, compound **2**

6. The ^{31}P-NMR spectrum of (5,10,15,20-Tetraphenylporphinato)dichlorophosphorus (V) chloride, compound 2

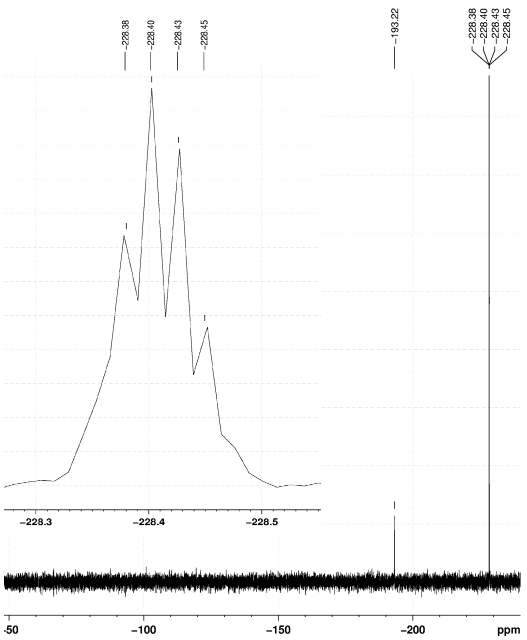

S6 The ^{31}P-NMR spectrum of (5,10,15,20-Tetraphenylporphinato)dichlorophosphorus (V) chloride, compound **2**

^{31}P-NMR (202.4 MHz, $CDCl_3$) δ: -193.22 (s, inner P(V) porphyrin, -228.4 (s, outer P(V)porphyrin)

7. HSQC ^{1}H-^{13}C and the HMBC spectra of (5,10,15,20-Tetraphenylporphinato)dichlorophosphorus (V) chloride, compound 2

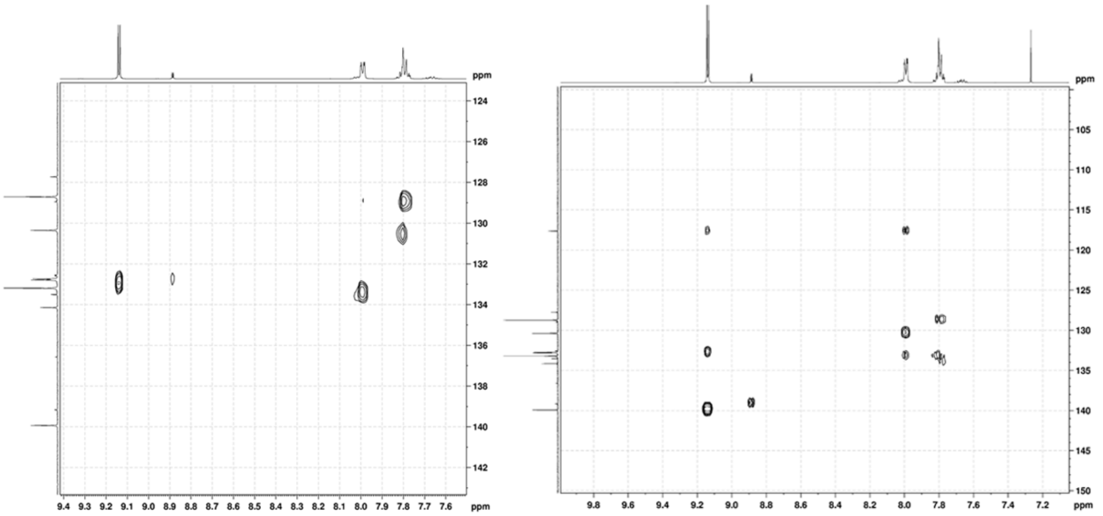

S7 The HSQC ^{1}H-^{13}C and the HMBC spectra of (5,10,15,20-Tetraphenylporphinato)dichlorophosphorus (V) chloride, compound **2**

8. Characterization of heterodimer complex compound 3

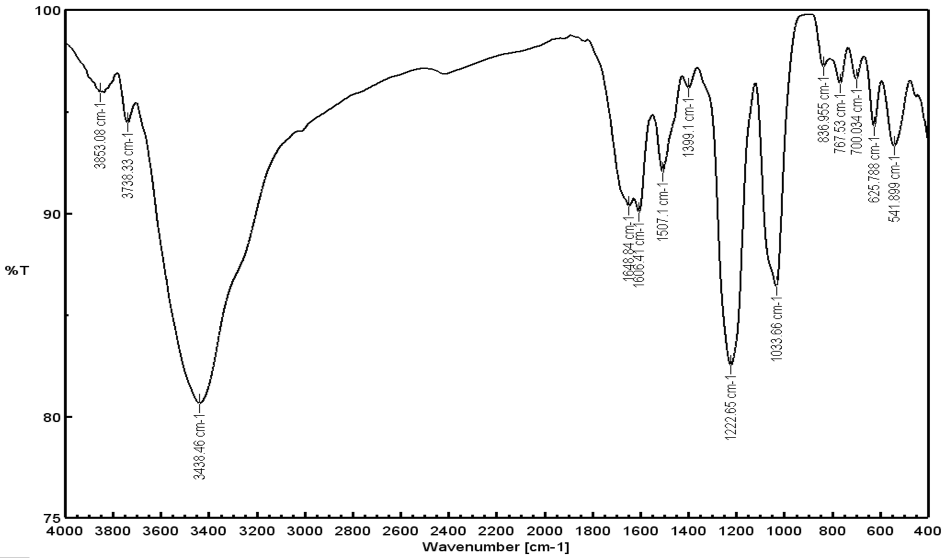

S8. FT-IR spectrum of dimer complex compound **3**

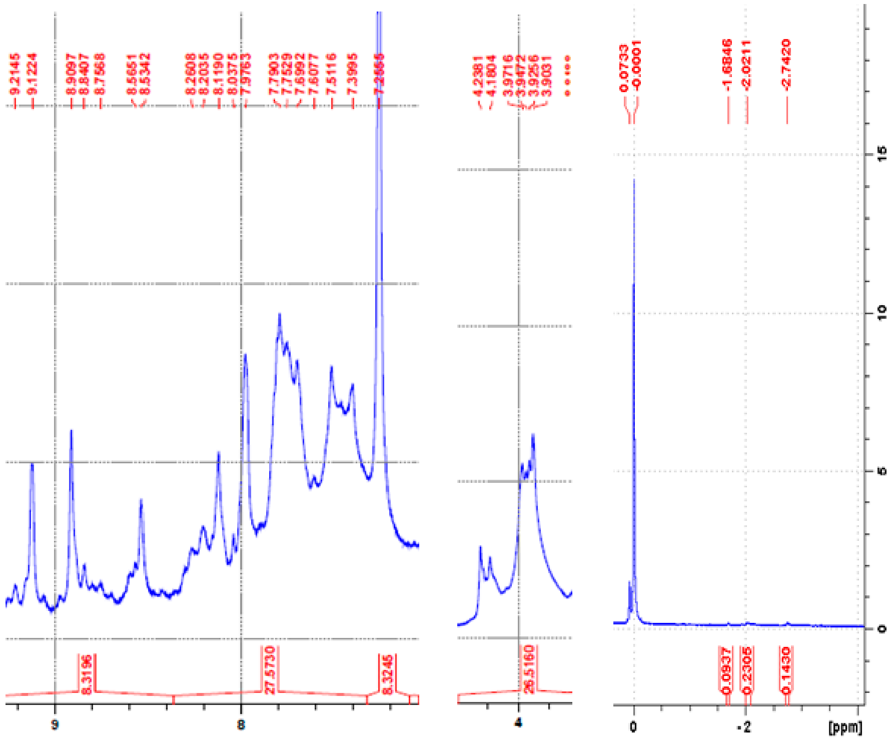

S9 ^{1}H-NMR and of the dimer complex compound **3**

This spectrum was introduced in the main manuscript as Figure 3

9. ^{31} P-NMR of the heterodimer complex compound 3

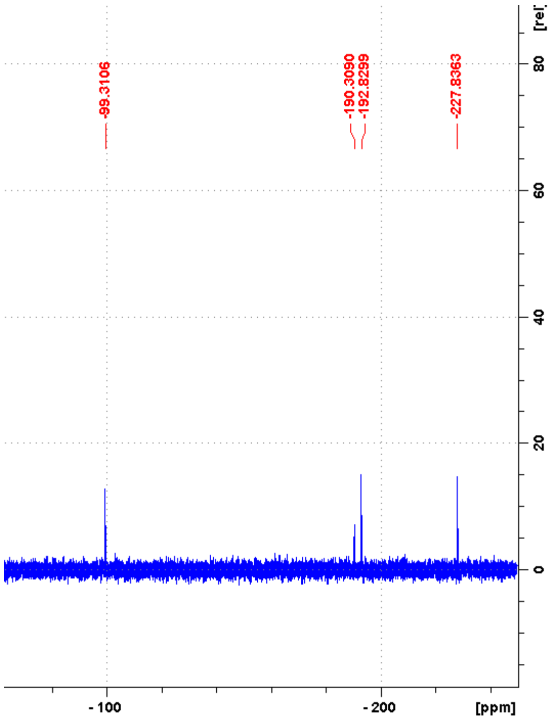

S10. ^{31} P-NMR of the heterodimer complex compound **3**

10. EDAX Quantification reveals, in strong agreement with NMR that to a Fe atom one P atom is corresponding.

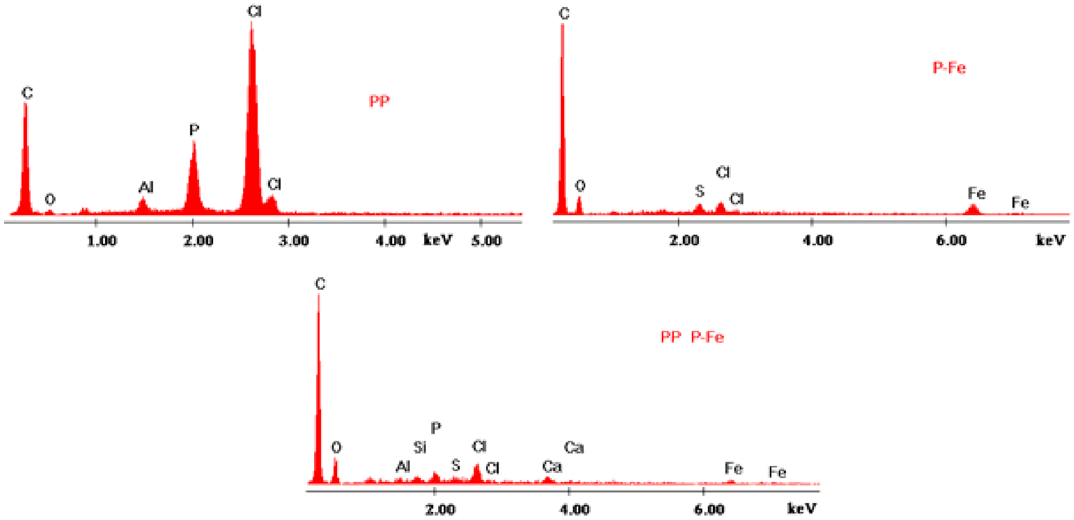

S11 EDAX Quantification of compound **2**, compound **1** and dimer complex compound **3**

11. UV-vis experiments between compound **3** and hydrogen peroxide solution

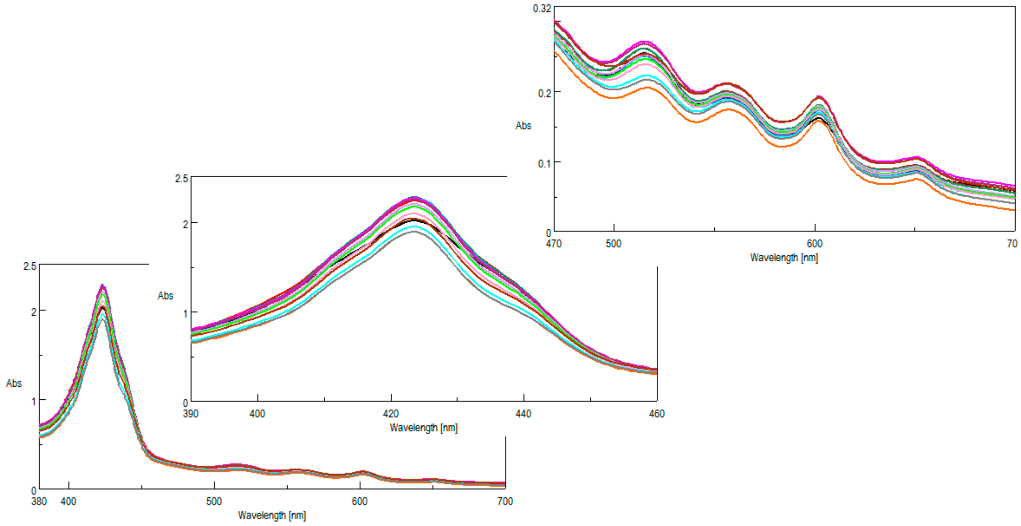

S12 UV-vis experiments between compound **3** and hydrogen peroxide solution. The decrease of the intensity of absorbtion by increasing the H_2O_2 concentration does not provide sufficient sensitivity.
